# Retraction: Insights into the progressive impact of high-fat-diet induced insulin resistance on skeletal muscle and myocardium: A comprehensive study on C57BL6 mice

**DOI:** 10.1371/journal.pone.0361831

**Published:** 2026-11-02

**Authors:** The PLOS One Editors

Following the publication of this article [1], concerns were raised with results presented in Figs 4 and 6, and the S1 File. Specifically,

- The following panels appear to (partially) overlap despite being used to represent different experimental conditions:
  - Fig 4 Con-8 TEM(8000×) (C2) and Fig 4 Con-10 TEM(15000×) (D3) when flipped.
  - Fig 4 Con-8 PAS(200×) (E2) and Fig 4 Con-10 PAS(200×) (E3).
  - Fig 6 Con-10 HE(400×) (B3) and Fig 6 Con-12 HE(400×) (B4).
  - S1 File skeletal muscle-PAkt-2 and S1 File skeletal muscle-PGSK3β-3.
- Contrary to the article’s Data Availability statement reading that all relevant data are within the manuscript and its Supporting Information files, the individual-level data from which the article’s graphs were prepared are not provided with the article.

The corresponding author stated that errors were made during the preparation of Figs 4 and 6 as well as the S1 File. They provided the original images for Figs 4 and 6 as well as updated versions of Figs 4 and 6, and the individual-level data underlying all figures presented in [1]. Editorial assessment of the individual-level data raised additional concerns about the integrity and reliability of these data.

In light of the above concerns, which call into question the reliability and integrity of the published results, the *PLOS One* Editors retract this article.

All authors did not agree with the retraction.
